# Utilization of Radium-Bead Material for Road Safety: An Application of the Circular Economy Concept

**DOI:** 10.3390/polym13213708

**Published:** 2021-10-27

**Authors:** Sajid Hussain, Xuemei Zhou, Syyed Adnan Raheel Shah, Naveed Ahmad, Muhammad Kashif Anwar, Muhammad Aamir Basheer

**Affiliations:** 1 Key Laboratory of Road and Traffic Engineering of the State Ministry of Education, Shanghai Key Laboratory of Rail Infrastructure Durability and System Safety, College of Transportation Engineering, Tongji University, 4800 Caoan Highway, Shanghai 201804, China; sajidbhatti1214@gmail.com; 2 Department of Civil Engineering, Pakistan Institute of Engineering and Technology, Multan 66000, Pakistan; Syyed.adnanraheelshah@uhasselt.be (S.A.R.S.); kashifanwar723@gmail.com (M.K.A.); 3 Department of Civil Engineering, University of Engineering and Technology, Taxila 47080, Pakistan; n.ahmad@uettaxila.edu.pk; 4 Center of Mobility and Spatial Planning (AMRP), Ghent University, B-9000 Ghent, Belgium; muhammad.basheer@ugent.be

**Keywords:** circular economy, road safety, radium beads (RPB), mechanical properties, reflecting materials, performance, sustainability

## Abstract

Road safety has become a serious issue in both developed and developing countries, costing billions of dollars every year. Road accidents at nighttime especially in low illumination situations are common and severe and have gained a lot of attention. To improve visibility and avoid traffic accidents, a series of efforts have been made but the existing mechanism is facing continuous challenges and highlighting a need for smart highways with high efficiency, road safety, and strength. In this study, the use of radium polymer beads (RPB) is proposed to avoid road accidents. The effect of RPB was investigated by comparing the results of the beads’ surface and modified asphalt mixtures using the three-stage testing methodology. Utilizing the circular economy, RPB have been introduced as a solution to the problem. Results indicated that in the first phase, the addition of RPB on the mixture surface improved the mechanical performance of the road pavement and helped in avoiding road accidents due to their ability to absorb the light from the source and then reflect in the night. Moreover, the mechanical properties using Marshall stability standard parameters (stability 9 kN and flow 2–4 mm range) were fulfilled as a standard testing requirement. The proposed radium bead layer can reduce road accidents and provide a direction towards future smart highways by using new reflective materials in road construction.

## 1. Introduction

Road safety has become a serious issue in modern countries, with road accidents costing billions of dollars in human and material expenses every year. With an ever-increasing population, traffic or road safety policies were introduced to preserve lives by preventing a surge in road fatalities [1]. Although it looks likely that the worldwide goal of halving traffic-related fatalities by 2030 will not be fulfilled [2]. More than 1.2 million people are predicted to die each year as a result of road accidents, while 50 million people are injured [3,4]. Users, cars, roads, and socioeconomic issues are all addressed in road safety policies [5,6].

Low illumination circumstances at night create frequent and major road accidents, and driving is constrained to stringent standards in aspects of the visual conditions [7,8]. Despite the fact that low street lighting is frequently installed on roadways at night, the line of vision for drivers is decreased due to restricted brightness and the reach of such illuminations. Consequently, most drivers do not reduce their speed appropriately to account for such visual limitations, boosting the dangers of night driving [7,9]. According to previous studies, the risk of an accident at night is 1 to 1.5 times greater than that of an event that happens during the day. Whereas the rate of road deaths per kilometer at night is nearly three times more than the daylight rate [10,11]. Hence, to develop more precise control mechanisms to assure the safety of drivers at night, a comprehensive analysis of the factors of severe nighttime road accidents in low-light situations is essential. Some previous studies have attempted to study the link between road conditions vs. safe driving at nighttime, focusing on the relation between lighting systems and traffic safety at nighttime. They found that the risk of traffic accidents was high during the night in comparison to the daytime [12,13]. In addition, lighting devices installed on the road can improve the visible range produced by headlights of vehicles, thereby reducing the number of fatalities and improving overall safety [14,15]. The risk of nighttime traffic accidents varies depending on the type of roadway. For example, metropolitan roads are less likely than rural roads to experience road accidents at nighttime [16]. Another interesting research study found that placing reflective road markers on the central axis of a curved section significantly minimized road crashes at nighttime, particularly single-vehicle fatalities [17]. Recently, glowing roads have been used as a road safety strategy in many developing countries. For example, a stretch of road in the Netherlands has been transformed into something straight out of a computer game. Road markings glow in the night rather than the typical white or yellow lane markers, alleviating the need for lighting [18].

**Figure 1:**
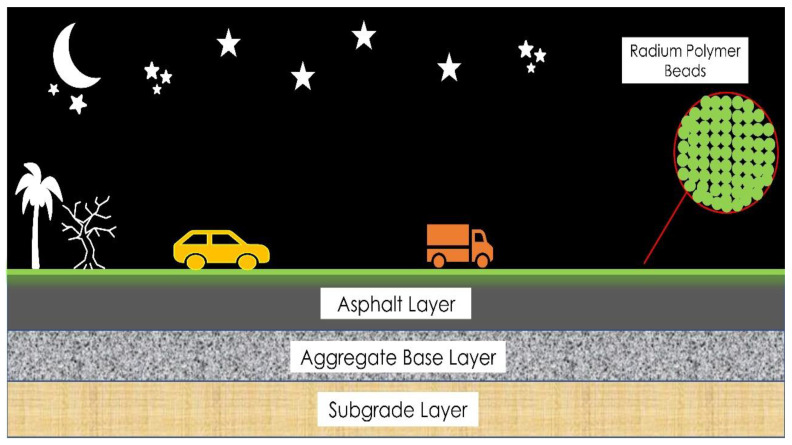
The proposed smart highways with radium polymer beads.

This study aims to introduce a smart road strategy by utilizing radium polymer materials as reflective material on the road surface to reduce the rate of traffic deaths at nighttime (Figure 1). Moreover, previous research has rarely considered the impact of accident features and road conditions. Therefore, these investigations are still primarily qualitative research; however, quantitative analysis is required to go deeper into the topic. Hence, this study addresses the shortcomings of roadways in terms of road performance as well as traffic accidents at nighttime due to the unavailability of traffic lights or in low illumination situations. The proposed radium bead layer can reduce road accidents and provide a direction towards smart highways for using new reflective materials in road construction.

## 2. Background

### 2.1. Radium Polymer

**Figure 2:**
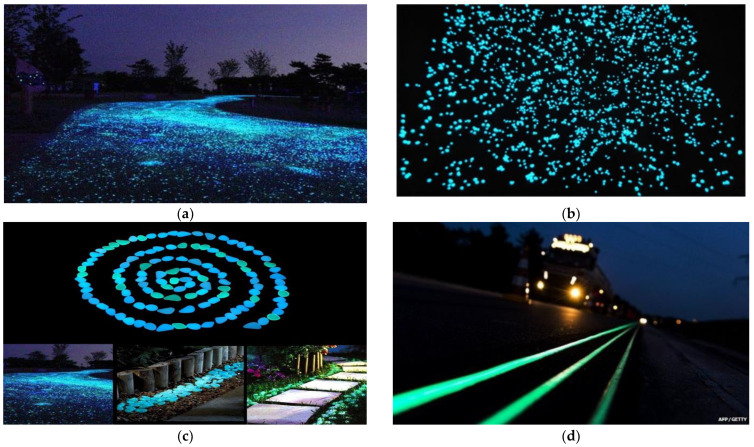
Different products used to illuminate the roadway at night [18]: (**a**) Luminescent product; (**b**) Core Glow products; (**c**) Unime pebbles; (**d**) photo-luminescent paint.

The utilization of luminescent materials on the surface of roadways is gaining popularity around the globe as shown in Figure 2. Roadways that glow in the dark, like a starry night, are not only attractive but also environmentally friendly. Luminescent materials can be utilized instead of expensive, energy-guzzling lights to brighten up driveways and sidewalks. For example, utilization of CORE Glow products, which are photo-luminescent stones that absorb light from the sun throughout the day and gradually emit their light at nighttime for up to 12 h. This product is available on the market in the form of recycled glass, marble, and plastic pebbles. Glittering pebbles, which are constructed of photo-luminescent pigment or resin, are also another readily available solution. Similarly, spray paint is also available and can be used to construct glow in the dark roadways made up of large stones. It can also be used for marking roadways or highways. During the construction of new roadways made from concrete, different pigments in combination with concrete materials can be added to provide an overall glow in the night.

The illuminating highways concept was first presented by Roosegaard in 2012 by a joint venture with Heijmans Infrastructure (Netherlands). Moreover, a stretch of road in the Netherlands has been transformed into something straight out of a computer game. Road markings glow in the night rather than the typical white or yellow lane markers, alleviating the need for lighting [18]. The idea was piloted on a 0.3-mile section of the N329 near Oss, which is situated southeast of Amsterdam. Photovoltaic photo-luminescent material is incorporated in combination with road-marking pigment, which recharges throughout the day. It can also glow on the driveway for up to 8 h at nighttime. The photo-luminescent pigment could enhance visibility on darker sections of roads or in regions where lane markings are difficult to see, thus minimizing the possibility of traffic accidents [18]. 

### 2.2. Circular Economy

**Figure 3:**
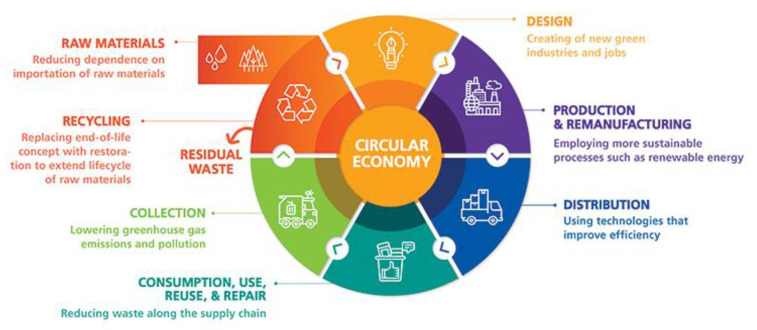
Circular economy model [28].

The circular economy (CE) is a strategy that tries to minimize waste production by preserving and fixing existing products and increasing the utilization of scrap materials while reducing the removal of virgin resources [19,20,21]. As a result, urban mining falls within this strategy, which aims to recover resources from all types of anthropogenic assets, including structures, infrastructure, and industries [22]. Poulikakos et al. [23] published a review paper that showed how a significant proportion of the waste generated in urban or peri-urban areas can be reused in road pavements [24]. The CE is defined in a variety of ways in previous literature [25,26]. The CE is defined as “a framework which can turn, conserve, and redistribute materials, products, and resources back into circulation in the most economically, functional, social, and effective possible way” [27]. Figure 3 shows the circular economy process, which explains the general development of cycles [28].

The selection of sustainable waste materials in roadways should be explored only if it is also ecologically superior to alternative usage in other applications, regardless of whether it is technically feasible or not. Presently, waste plastics have become a global issue [29]. Numerous waste products, such as glass, bitumen, wood, waste tires, waste car bumpers, and various forms of plastic waste, were proven to have a technical potential for reprocessing in asphalt pavements [30].

One of the most effective ways to improve the overall performance of an asphalt mixture is to replace it with polymers. However, the cost-effectiveness and carbon impact of polymer modification versus overall performance has sparked a hot discussion among various pavement researchers [31,32]. Polymer additives made from recovered waste materials have gained considerable interest from scholars due to their dual advantages of cost-effectiveness and addressing the issues for waste disposal from an ecological and financial aspect [33]. As a result, one of the potential choices in urban pollution control might be the effective utilization of plastic materials in asphalt mixtures. When it comes to stability performance, polymers or plastics in asphalt mixtures have problems related to their homogeneity. Phase separation has been identified as a prevalent issue in several investigations [34,35,36,37]. Different materials can be used for this purpose, as composite materials can help in the production of flexible materials [38,39], even prolonged temperature has an impact on this type of production [40,41] and sometimes meta-analysis is also conducted to analyze in-depth before recommending different materials [42].

The application of the circular economy concept has improved the economical construction process of construction and now its connection with safe and efficient infrastructure development will improve the future of the industry. This study will testify to the proposed phenomenon of the utilization of radium beads as a low-cost energy-efficient solution.

## 3. Materials and Methods

**Figure 4:**
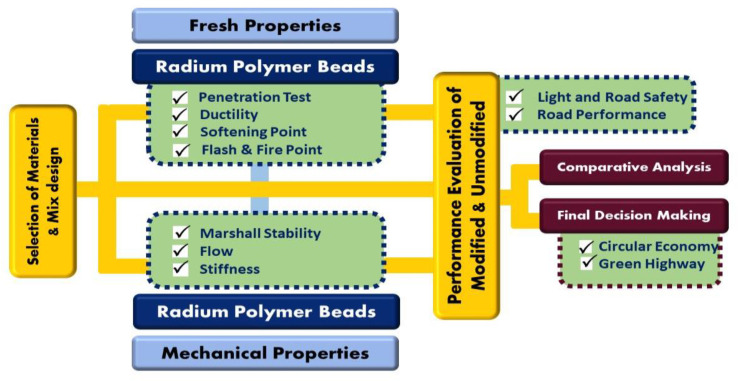
Complete research methodology (flow chart).

The research methodology is divided into two phases. In the first phase, the role of radium polymer beads was studied in terms of both road safety and road performance. In the second phase, an asphalt concrete mix was modified with the help of the same material to study an in-depth analysis of their mechanical characteristics such as stability, flow, indirect tensile strength (ITS), and stiffness. The comprehensive research methodology adopted in this study is shown in Figure 4.

### 3.1. Basic Materials

**Figure 5:**
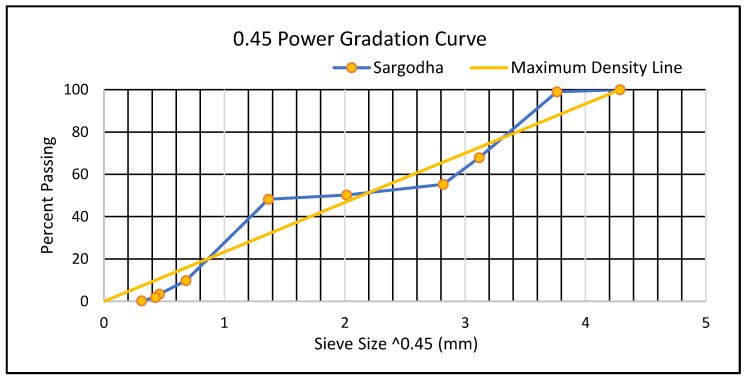
Particle size distribution curve for utilized aggregates (Sargodha type).

Asphalt binder 60–70 grade with a penetration value of 65 mm was used as a base binder (reference) for radium polymer modification (RPM). The pure binder was replaced with polymeric material designated as green radium beads (also known as radium acrylonitrile butadiene styrene (ABS) granules) which were obtained from the local market in Pakistan, consisting of source material imported from the Advanced Petrochemical Company (Saudi Arabia) [43]. Millions of tons of these materials are produced by their factories around the world. The beads glow in a dark environment and are used locally as ornaments. This polymeric material is suitable for use as a binder modifier. The utilization of radium polymer beads as a bitumen binder could be extremely helpful in terms of potential substitutes, cost benefits, and increased pavement performance. Likewise, the use of radium beads on the road surface will also help to reduce the risk of road accidents at night. The basic fresh properties of both modified polymers and unmodified bitumen were measured using ASTM standards and the results are summarized in Table 1 along with their standard range and specifications. The physical characteristics of both aggregates used are also summarized in Table 2. A sieve analysis test was conducted for better selection of both aggregates used (fine and coarse) and their distribution curve and is presented as shown in Figure 5.

According to the grading curve that was generated by the ASTM Standard C136, the resulting curve is in the gap graded categories which implies that not all particle sizes are present in the aggregate sample, thus this rests on the stone mastic asphalt (SMA). This type of aggregate is based on the 0.45 power gradation chart of the Federal Highway Administration (FHA), indicating that the grading line should stay away from the maximum density to enhance the voids in mineral aggregates (VMA). This is done so since the surface area is also expanding owing to the maximum density line, which leads to additional bitumen needs. Plastic deformation and rutting also happen because of the high proportion of bitumen. The most vital thing after selecting the proper aggregates or binder is to arrange aggregate particles according to the grading standards prescribed in the specifications. In this study, the 0.45 power gradation chart from the Federal Highway Administration (FHA) was used for the preparation of asphalt mixtures. To maximize the VMA, it is recommended that the gradation line should be kept away from the maximum density line (MDL) due to following reasons: (i) The maximal surface area will be achieved if the aggregate gradation follows the MDL; (ii) the need for bitumen will rise because of the increased surface area, requiring the consumption of more bitumen; (iii) the mix contains more bitumen content, and it will compact under load conditions, which ultimately result in plastic deformation (rutting); (iv) moreover, the number of voids (gaps) in mineral aggregate (VMA) and pore spaces will also be reduced.

### 3.2. Radium Polymer Beads

The radium beads were obtained from the market in the form of beads known as radium acrylonitrile butadiene styrene (ABS) granules. In this study, the radium polymer beads used had an average particle size of 7.5 mm. These beads were utilized in two forms. First, they were used on the surface of the asphalt concrete mix to take advantage of its glittering phenomenon, as a glittering road will help in road safety. Second, these beads were used as binder modifiers. They could not be mixed homogeneously with the hot bitumen (binder), that is why the beads were grinded to smaller sizes before being mixed with the binder to make the process easier and increase the quality of the blends in terms of homogeneity. This process was performed to utilize beads as a binder and to mix it with the primary binder (bitumen). The size of the particles after grinding was reduced from 7.5 mm to a range of 1 mm–0.075 mm. It was easy to mix particles of these sizes with the binder along with heating. The physical and chemical properties of the radium beads are expressed in Table 3, showing a potential material available for pavement construction.

### 3.3. Blending Process and Preparation of Radium Polymer Samples

The mixing process is dependent upon several factors such as mixing time, temperature conditions, mixer speed as well as mixing methods: wet or dry. In addition, previous research has demonstrated that these criteria varied depending on the study’s nature. There is no universal agreement on such factors, as scholars have utilized diverse methods depending on their expertise. As a result, the mixing parameters of a polymer asphalt mixture are selected based on personal preferences and experiences. In this study, the wet method was chosen for the blending process. Five different percentages ranging between 0 to 10% were chosen and replaced with bitumen content by the total weight of the binder. At first, 60–70 binder was heated then shredded pieces of radium polymer were added according to the corresponding percentage. This was performed by removing the equivalent amount of OBC by the weight of bitumen material (grams) according to each polymer-substituted percentage. Before testing, trial samples at different percentages of binder content were prepared to find the optimum binder content (OBC) which was measured as 7% in this study. For homogenous mixing, both polymer content and pure binder was mixed properly for about 5 to 10 min. Similarly, the aggregate was heated between 150 to 170 °C. After that, bituminous materials were heated together to achieve homogeneity. The modified and unmodified polymer specimens were prepared at different percentages for comparative analysis of the results. In this study, three samples of each dosage were prepared, and then average readings were taken.

Similarly, the radium polymer beads were also placed on the upper and lower surface of samples for Marshall stability analysis and flow. In this study, beads were placed on both sides of the samples for road safety purposes. The beads will glow at night as they are luminescent by nature. The idea behind this concept was to introduce smart roads, which communicate with drivers at night when there is no traffic light available on the roads or highways.

## 4. Results and Discussions

### 4.1. Data Analysis

**Figure 6:**
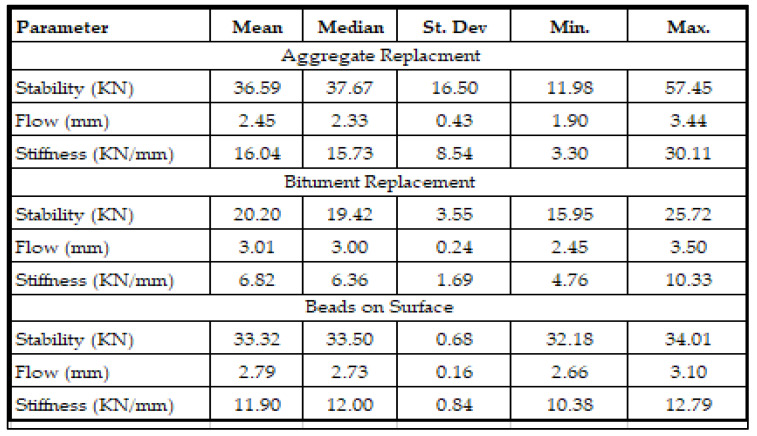
Statistical data description of performance properties of asphalt concrete mix.

All the properties of modified asphalt mixtures were statistically analyzed such as Marshall stability, Flow, Indirect tensile strength (ITS), and Stiffness. The Excel statistical program was used to determine the data description of stability, flow, and other characteristics of the modified and unmodified core samples. The results of the data analysis which included descriptive data analysis, mean, mode, median, variance, and standard deviation are summarized as shown in Figure 6.

### 4.2. Physical Performance of Polymer Modified Binder

**Figure 7:**
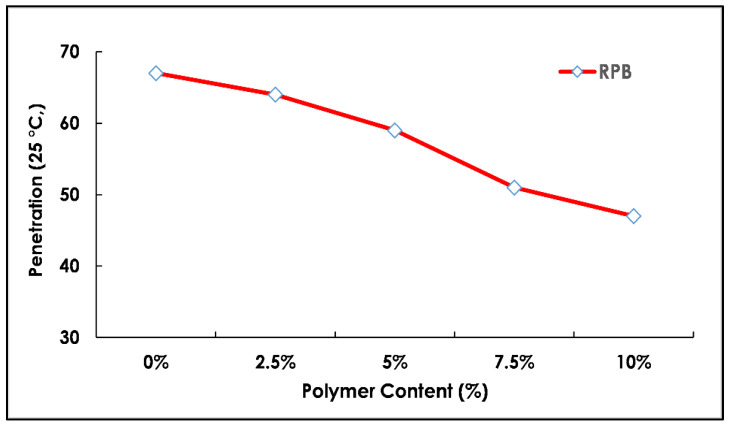
Penetration test results with varying polymer content (%).

**Figure 8:**
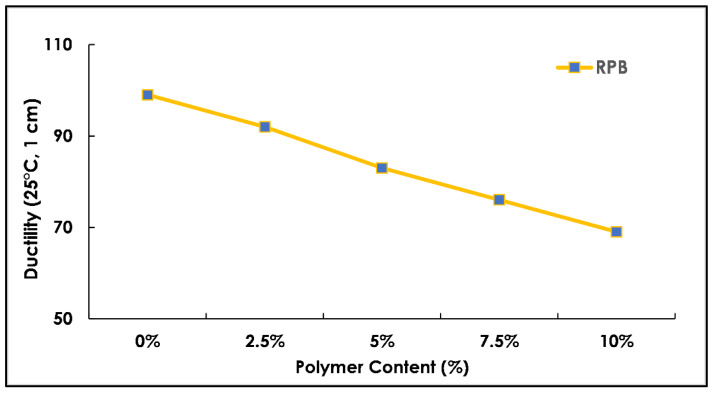
Ductility test results with varying polymer content (%).

The properties of the pure and modified binder with radium polymer materials were comprehensively studied for an in-depth analysis of the results as shown in Figure 7 and Figure 8. Four basic tests of the conventional binder such as softening point (°C), flash point (°C), ductility (@ 25 °C), and penetration tests (0.1 mm) were conducted. The test methods used in this study were all standard for measuring the effects of radium polymer addition in the pure binder. The results of polymer-modified bitumen were then compared with unmodified (reference) bitumen for a better understanding of binder behavior upon polymer addition. Five different percentages of radium polymer materials 0–10% were selected and replaced binder 60–70 by weight of total optimum binder content, which was 7% in this study.

A penetration test refers to the measurement of standard consistency of asphalt binder in which a standard needle penetrates the specimen. The penetration test was conducted for both types of bitumen (modified and unmodified). The penetration results are presented in Figure 7. According to laboratory results, a decrease in the penetration reading was observed with increasing radium polymer content. During the blending process, it was noticed that modified bitumen was not completely homogenous, as the radium polymer material could not be mixed properly within a pure binder and remain suspended at the top layer which might change the results. The penetration test was performed after the milling of the top surface and results are displayed in Figure 7. Moreover, it was observed that the average penetration values fall with an increase in polymer contents of up to 10%. This might have happened due to strong adhesive bonding between the modifier and binder. Therefore, polymer modified binder is recommended in hot regions because in such regions low penetration values were ideal to avoid softening of the binder. In conclusion, it is recommended to practice in hot climate zones and where there is a heavy traffic load.

The ductility test was conducted as per ASTM standard. In this test, the binder was elongated at a speed of 5 cm/min at a testing temperature of 25 °C. The breaking of the specimen was recorded and called ductile value. The ductility results of both modified and unmodified bitumen are presented in Figure 8. It was observed that the ductility reading decreases with an increase in polymer content at varying percentages of 0–10%. The standard limit of the ductility value was >75. The decrease in ductility values may be due to weak bonding between the aggregate and binder. The ductility property is also the result of the binder performance and if a physically harder material is mixed with a binder-like bitumen it reduces its ductility property, which is why a low percentage of modification is recommended, which creates a control on ductile behavior. During the continuous loading condition, if a material does not show ductility, it tends to break within a certain limit due to the repetition of loads. In the case of road material, a ductile material is recommended, and modification of bitumen is also recommended up to a certain limit.

**Figure 9:**
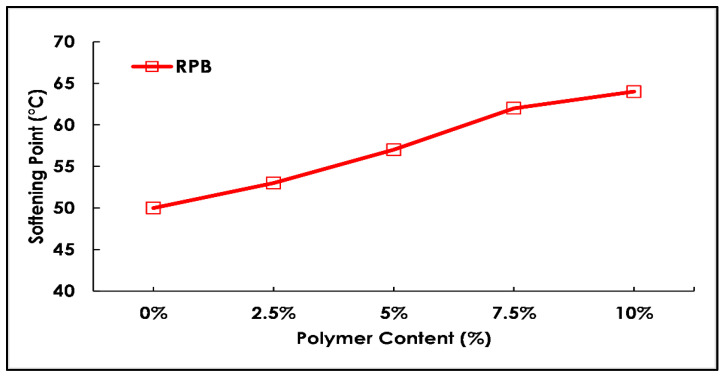
Softening test results with varying polymer content (%).

Bitumen is a viscoelastic material by nature and its melting point is high. It becomes softer with an increase in temperature. In this study, a softening point (Ring & Ball) test was performed on a pure binder as well as a modified binder containing a varying percentage of polymer materials. The results of the softening point are displayed in Figure 9. As per laboratory results, it was seen that softening point values for the modified binder were increased with the increasing percentage level of polymer content (0 to 10%). The test results support the discussion to be used in hot climate areas. In conclusion, the polymer material increases the softening point values (softening temperature) and thereby enhances overall stability under high-temperature conditions.

**Figure 10:**
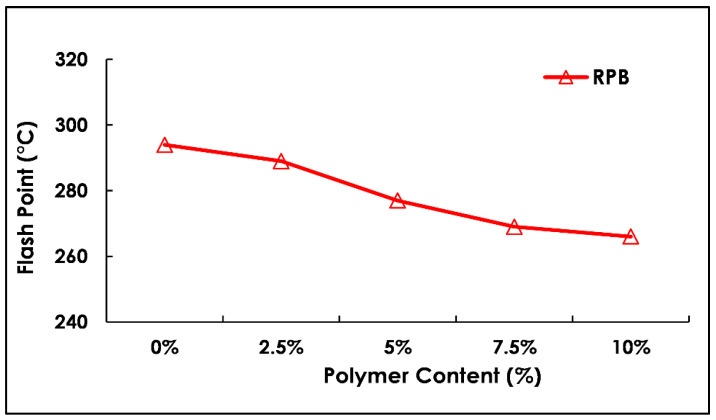
Flash point test results with varying polymer content (%).

The result of the flashpoint test is displayed in Figure 10. It was observed that the flashpoint reading decreases with an increase in polymer contents. The maximum value was recorded at 0% (control sample) whereas after this reading tends to fall up to 10% replacement of binder with polymer material. The standard minimum value is 232 as per the ASTM test method. In this study, the flashpoint readings were greater than the control sample.

### 4.3. Marshall Stability—Flow Analysis

**Figure 11:**
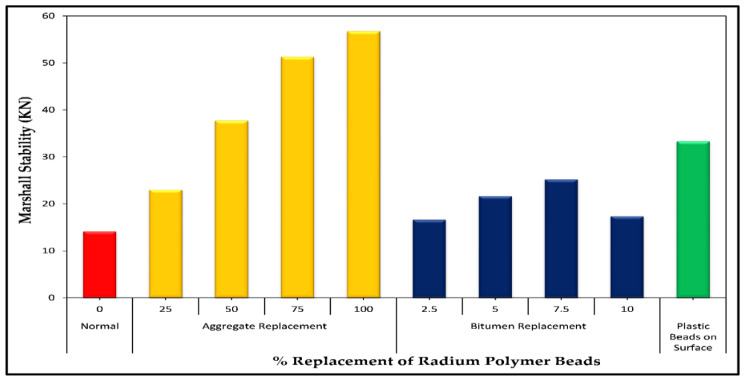
Marshall stability versus radium polymer materials (%).

**Figure 12:**
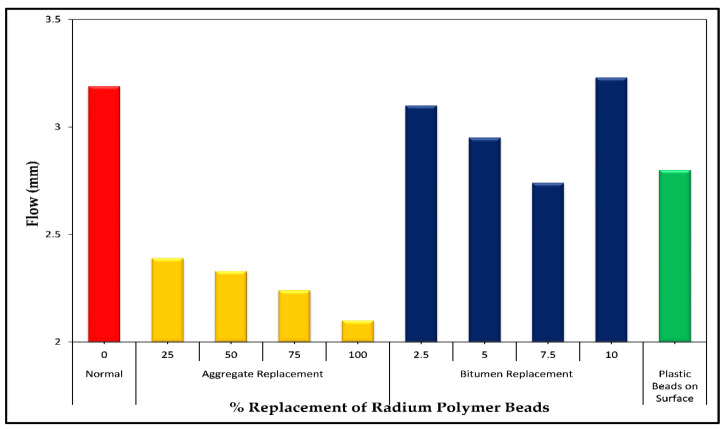
Marshall flow versus radium polymer materials.

**Figure 13:**
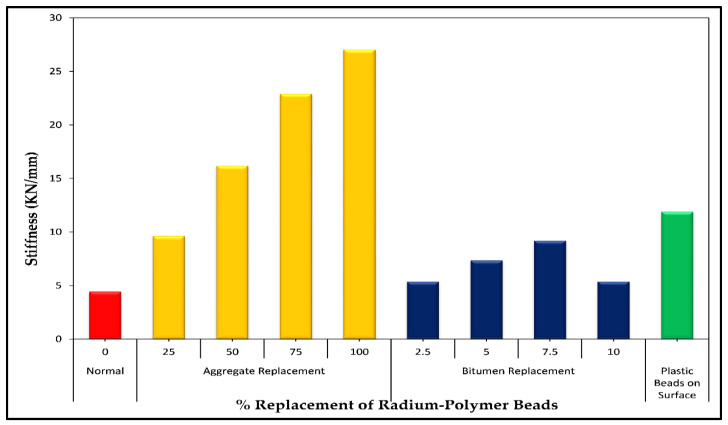
Stiffness versus radium polymer materials (%).

The Marshall testing was simply carried out to study the performance of road pavements such as failure or rutting under the influence of a heavy traffic load. The mechanical properties of stability, flow and stiffness are presented in Figure 11, Figure 12 and Figure 13, respectively. It is clear from Figure 11 that the stability of modified asphalt concrete was enhanced with an addition of polymer material up to 8%. Hence, polymer-based asphalt mixtures showed better stability (strength) as compared to the control specimen. The Marshall test method is a widely used indicator for the assessment of bituminous materials susceptibility to deformation and ability to withstand substantial traffic loads [50,51,52]. According to the standard, a minimum of 9 KN stability is the requirement for wearing layers. In this study, the stability of polymer-based mixtures was increased by up to 7.5% of radium polymer replacement. After that slight change instability, the value was observed at a 10% replacement level.

On the other hand, the aggregate of #4 sieve was also replaced with four different percentages of a radium polymer material such as 0%, 25%, 50%, and 100%. The Marshall stability was checked for each percentage. It is clear from Figure 11 that the stability of polymer asphalt mixtures was enhanced with the addition of polymer as an aggregate replacement.

The reason is that during preparation when aggregates were heated in combination with polymer beads, which made the strong bonding between aggregates and pure binder. The samples with radium polymer on both surfaces (upper and lower) were also prepared and their stability was checked in the Marshall stability machine. According to the results, the stability of asphalt mixtures with polymer addition on surfaces was improved. The flow values of the polymer-modified asphalt mixtures decreased with increasing polymer content up to 7.5% as shown in Figure 12. This could be due to the stiffness of the RPB-modified mixtures. The maximum flow of 3.23 was observed at 10% replacement of radium polymer materials, which is still within the standard range (2–4 mm). The Marshall flow test can be used as an indicator for rutting resistance. It was clear from the results that the addition of RPB (polymer materials) in asphalt mixtures helped to improve its resistance against deformation. Consequently, the addition of polymer materials improved the stability of asphalt mixtures whereas, utilization of such materials affected flow values and these findings are consistent with earlier studies [53,54]. Similarly, the resistance against deformation was improved with the addition of polymer beads on both faces of the specimen, which was 2.8 mm, and it was 12.23% less when compared with control asphalt mixtures.

### 4.4. Stiffness

The stiffness (modulus) of the road pavement is the measurement of the structural performance of the road layer to the pavement’s strength or durability. It is the only parameter that is directly related to the thickness design method of the road pavement. The road pavement would be more durable or stronger if the asphalt mixtures were stiffer. Similarly, a thinner pavement can be stiffer without affecting its load-bearing capacity. The stiffness parameter is not directly measured because Marshall stability and flow are considered as the measurement of the binder’s contribution in bituminous mixtures. The results of stiffness (stability/flow) are shown in Figure 13. From the bar graph, it was observed that modulus values of polymer-modified bituminous mixtures are higher as compared to the control mixture. The continuous increase in stiffness values was measured as the percentage of radium polymer increased up to 7.5% and which is the highest value (9.19 KN/mm). This value then dramatically falls to 42% with the addition of more polymer material i.e., 10%.

The increase in stiffness values was recorded in the case of polymer addition for each percentage of aggregate replacement with sieve #4. The addition of polymer-modified mixtures had higher stiffness values which indicated more resistance against permanent deformation [55]. The mean stiffness value for control samples was 9.63 (KN/mm). The radium polymer beads increased the stiffness values at 25%, 50%, 75% and 100% replacement of sieve #4 aggregates by 54%, 40.57%, 57.97% and 64.36%, respectively. Similarly, increased stiffness values were observed when polymer beads were placed on the surface of asphalt mixtures, which was 62.78% stiffer as compared to control asphalt mixtures.

The wet approach was recommended by the researchers in most trials for polymer modification; this can be related to having a better handle on the binder’s effectiveness before putting it in place on the site. To lessen the gap between radium polymer and asphalt binder, additives or improvements to the modified surface qualities are required to be substituted partially i.e., more than 7.5%. The effects of the polymer addition in asphalt mixtures on the performance of road materials in terms of other mechanical tests such as wheel tracking tests, rutting, and its effects on human health need to be studied in the future. This research also points to the future directions towards the collection and application of radium polymer beads in road construction as well as waste management. The effects of employing various reflective materials (various types of polymers) on the parameters of the modified binder and road performance must be better understood and controlled. Moreover, this research will support the utilization of more polymer materials as luminescent materials. It is necessary to make wise decisions when implementing new techniques and to evaluate the outcomes of each action in terms of investments, risks, and yields, as well as to ensure the sustainability of road construction projects.

## 5. Conclusions

The present study evaluated the effect of the polymer addition on the overall performance of the asphalt pavement. In addition, the potential utilization of polymer materials was studied for the enhancement of the road pavement as well as road safety purposes. Radium polymer materials as reflective materials have positive outcomes in terms of improving road safety in low illumination circumstances. It was observed the addition of polymer content in combination with a pure binder significantly improved the mechanical performance of polymer-modified bitumen in terms of Marshall stability and increased stiffness. The increased stability values were recorded with an increase in polymer content up to 7.5%. It was observed that the polymer modified asphalt mixture showed superior strength as compared with the conventional binder 60–70. Moreover, the radium polymer could help to improve overall road performance. It is evident from fresh properties of both modified and unmodified bitumen that utilization of polymer materials in pure binder reduces the penetration and ductility readings whereas, softening point values were increased. Strict guidance, regulation, and support to promote the use of luminescent materials by contractors in the construction and restoration of road pavement are needed, particularly in developing countries for road safety purposes. Managing traffic authorities should put in place appropriate road safety mechanisms and support the smart highways concept to reduce the risk of traffic accidents and to effectively handle this challenge. The use of radium polymer as a bitumen modifier as well as for road safety will help to provide positive outcomes in terms of both improved performance and reduced traffic accidents caused by the unavailability of streetlights on the roads or highways.

The utilization of radium polymer contents requires a suitable mixing method as well as a blending machine to optimize the proper mixing of both polymer and binder content. Therefore, special attention should be paid in this regard. Polymer modified asphalt mixtures may not have an adverse influence on low temperature cracking characteristics of bituminous mixtures if it is properly prepared. Moreover, to improve the interaction between the polymer and bitumen, it is required to choose low-cost additives.

## Figures and Tables

**Table 1:** Basic properties of binder used for asphalt mix concrete.

Property	Results	Limit	ASTM Standard
Ductility 25 °C (cm)	99	>75	ASTM D113 [44]
Softening Point (°C)	50	40–55	ASTM D36 [45]
Penetration @ 25 °C (0.1 mm)	67	60–70	ASTM D5M-20 [46]
Flash Point (°C)	294	232 min	ASTM D92-18 [47]

**Table 2:** Physical characteristics of utilized aggregates for asphalt mix concrete.

Parameters	Results	Range	Standard
Coarse Aggregates			
Water Absorption	1.60%	<2%	ASTM C127 [48]
LA Value	28%	<35%	ASTM C131
Aggregate Impact Test	24.3%	<27%	BS812 Part3
Specific Gravity	2.43	2–3	ASTM C127 [48]
Fine Aggregates			
Water Absorption (%)	0.62%	<2	ASTM C128
Soundness Test	7	<15	ASTM C88 [49]

**Table 3:** Physical and chemical properties of radium beads.

Chemical Properties	
Chemical Composition	Radium ABS Granules (Beads)
Physical Properties	
Density	545.95 Kg/m^3^
Specific Gravity	0.546
Melting Point	170 °C
Diameter	7.5 mm
Weight	0.216 g
Contraction (%)	0.005%

## Data Availability

Data will be available on suitable demand.
